# Rapid and Sensitive Detection of Water Toxicity Based on Photosynthetic Inhibition Effect

**DOI:** 10.3390/toxics9120321

**Published:** 2021-11-26

**Authors:** Min Chen, Gaofang Yin, Nanjing Zhao, Tingting Gan, Chun Feng, Mengyuan Gu, Peilong Qi, Zhichao Ding

**Affiliations:** 1 Key Laboratory of Environmental Optics and Technology, Anhui Institute of Optics and Fine Mechanics, Hefei Institutes of Physical Science, Chinese Academy of Sciences, Hefei 230031, China; minchen7@mail.ustc.edu.cn (M.C.); njzhao@aiofm.ac.cn (N.Z.); ttgan@aiofm.ac.cn (T.G.); cfeng@aiofm.ac.cn (C.F.); mygu@aiofm.ac.cn (M.G.); plqi@aiofm.ac.cn (P.Q.); zcding@aiofm.ac.cn (Z.D.); 2 University of Science and Technology of China, Hefei 230026, China; 3 Key Laboratory of Optical Monitoring Technology for Environment of Anhui Province, Hefei 230031, China

**Keywords:** photosynthetic inhibition, photosynthetic fluorescence parameter, *Chlorella pyrenoidosa*, toxic water pollutants, rapid detection, biotesting

## Abstract

To achieve rapid and sensitive detection of the toxicity of pollutants in the aquatic environment, a photosynthetic inhibition method with microalgae as the test organism and photosynthetic fluorescence parameters as the test endpoint was proposed. In this study, eight environmental pollutants were selected to act on the tested organism, *Chlorella pyrenoidosa*, including herbicides (diuron, atrazine), fungicides (fuberidazole), organic chemical raw materials (phenanthrene, phenol, p-benzoquinone), disinfectants (trichloroacetonitrile uric acid), and disinfection by-products (trichloroacetonitrile). The results showed that, in addition to specific PSII inhibitors (diuretic and atrazine), other types of pollutants could also quickly affect the photosynthetic system. The photosynthetic fluorescence parameters (Fv/Fm, Yield, α, and rP) could be used to detect the effects of pollutants on the photosynthetic system. Although the decay rate of the photosynthetic fluorescence parameters corresponding to the different pollutants was different, 1 h could be used as an appropriate toxicity exposure time. Moreover, the lowest respondent concentrations of photosynthetic fluorescence parameters to diuron, atrazine, fuberidazole, phenanthrene, P-benzoquinone, phenol, trichloroacetonitrile uric acid, and trichloroacetonitrile were 2 μg·L^−1^, 5 μg·L^−1^, 0.05 mg·L^−1^, 2 μg·L^−1^, 1.0 mg·L^−1^, 0.4 g·L^−1^, 0.1 mg·L^−1^, and 2.0 mg·L^−1^, respectively. Finally, diuron, atrazine, fuberidazole, and phenanthrene were selected for a comparison of their photosynthetic inhibition and growth inhibition. The results suggested that photosynthetic inhibition could overcome the time dependence of growth inhibition and shorten the toxic exposure time from more than 24 h to less than 1 h, or even a few minutes, while, the sensitivity of the toxicity test was not weakened. This study indicates that the photosynthetic inhibition method could be used for rapid detection of the toxicity of water pollutants and that algae fluorescence provides convenient access to toxicity data.

## 1. Introduction

Due to the rapid development of industry and agriculture, the types and quantities of pollutants discharged by human beings into the environment are increasing, and the resulting water pollution problems are becoming more and more serious. At the same time, the ecotoxicological data of these pollutants is seriously lacking [1,2]. Conventional physical and chemical detection methods can only quantitatively analyze the content of toxic pollutants and cannot reflect the comprehensive biological effects of toxic pollutants on the environment. As an important means of evaluating and predicting toxic pollutants, biological toxicity detection can intuitively and comprehensively reflect the comprehensive toxicity of pollutants to biological populations, synthesize the interaction between different toxic pollutants, determine the relationship between the concentration of toxic pollutants and biological effects, and provide a theoretical basis for environmental monitoring and a comprehensive evaluation of pollutants [3]. Common test organisms for biological toxicity monitoring include fish [4], photobacteria [5], invertebrates [6], and algae, etc. These biomarkers range from early biomass, to later physiological and behavioral changes, such as predatory behavior [7], phototaxis [8], and metabolic processes [9]. Compared with other tested organisms, microalgae are primary producers, and all its changes in the water environment will eventually affect the structure and function of the aquatic ecosystem. Furthermore, microalgae have a short culture cycle, simple operation, easy observation, small size, and sensitivity to toxic substances [10]. Therefore, microalgae are often used as ideal test organisms in aquatic toxicity testing.

The algae growth inhibition method, using biomass as the test index, has been standardized by the Organization for Economic Cooperation and Development [11] and the US Environmental Protection Agency [12] for chemical toxicity testing. As a group-based toxicity test index, growth inhibition does not give information about single cell response, which may lead to weak timeliness of response to the toxicity. By contrast, the photosynthesis process is population-independent and highly sensitive to the presence of toxicants, which leads to their increased use as ecotoxicological endpoints. Consequently, plant biochemical parameters linked to photosynthesis, such as adenosine triphosphate (ATP) formation [13], CO_2_ fixation [14], and O_2_ evolution [15], have been used as indicators for the effect of environmental stresses. However, the complexity of these methods and their strict detection environment rule out their use as convenient tools in environmental studies.

The fluorescent properties of the chlorophyll of living algae are a powerful tool to evaluate the photosynthetic performance of algae and analyze their protection response [16,17]. Through fluorescence dynamics technology, a complete set of biological information of the photosynthetic system II can be obtained, and there are different parameters corresponding to different processes of electron transport in photosynthesis. In addition, the rapid, simple, sensitive, and non-invasive characteristics of fluorescence kinetics technology give photosynthetic inhibition methods based on photosynthetic fluorescence parameters the potential to achieve rapid and real-time measurement of comprehensive water toxicity. A number of studies have shown that photosynthetic fluorescence parameters can be used as early indicators of the toxicity of water pollutants [18,19,20,21,22]. However, most of these studies focused on the application of single photosynthetic fluorescence parameters (Fv/Fm) in ecotoxicology and did not make good use of other photosynthetic fluorescence parameters for toxicity evaluation. In addition, the toxicity exposure time of photosynthetic inhibition was not further optimized.

Different algae have different sensitivity to toxicants, and there is no possibility of choosing an algae species that is the most sensitive to all toxicants. Based on a consideration of the particular situation, algae species which are easy to cultivate and evenly distributed should be the first choice. In terrestrial freshwater ecosystems, *Chlorella pyrenoidosa*, a common freshwater algae species, which is sensitive to toxicants, fast reproducing, small in size, widely distributed, and easy to obtain, is very suitable for studying the toxicity of environmental pollutants. In this study, *C. pyrenoidosa* was used as the test organism, and photosynthetic fluorescence parameters were used as the end point of toxicity detection. A series of rapid toxicity detection and quantitative analyses of different water pollutants were carried out under controlled conditions. Furthermore, comparing the results with the traditional growth inhibition method, a fast, sensitive, non-invasive method for detecting and evaluating water toxicity was proposed.

## 2. Materials and Methods

### 2.1. Algal Cultures

*C. pyrenoidosa* (FACHB-5) was selected as the test organism and was purchased from the Freshwater Algae Culture Collection at the Institute of Hydrobiology, Chinese Academy of Sciences (Wuhan, China). BG11 medium and 1 L Erlenmeyer flasks for inoculation and culture of *C. pyrenoidosa* were sterilized by autoclave for 30 min. After aseptic inoculation, Erlenmeyer flask cultures were incubated at 25 ± 1 °C in a constant temperature shaker incubator (MQD-S3R). A white cold-fluorescent tube was used as light source with an automated light/dark cycle of 12 h/12 h, and light intensity 120 μmol (m^2^·s)^−1^. All flasks were shaken 2 times daily for 15 min with speed of 120 r·min^−1^. Before the experiment, the algae were incubated for 3–4 days to reach the exponential growth phase [23]. During the cultivation process, a phytoplankton fluorescence meter (FluoroProbe, Schwentinental, Germany BBE) was used to monitor the change of the algal chlorophyll concentration. When the algae culture reached the medium logarithmic phase, the algae suspensions were diluted to 100 μg·L^−1^ chlorophyll concentration with BG11 medium for toxicant exposure.

### 2.2. Toxicants and Exposure Testing

In order to simulate different types of polluted water bodies, we tested 8 common organic pollutants in water, including herbicide (atrazine, diuron), fungicide (fuberidazole), organic chemical raw materials (phenanthrene, p-benzoquinone, phenol), and disinfectant and its by-products (tricloacetonitrile uric acid, trichloroacetonitrile), as shown in Table 1. Since most organic compounds were insoluble in water, dimethyl sulfoxide (DMSO) was used as a solvent to treat water-insoluble toxicants, in order to prepare pollutant stock solutions. Finally, a small volume (<1% culture volume) of the toxicant solution was added to the 50 mL algae suspension, while the DSMO content in the final sample was less than 0.1%. The pre-experimental results with OD_680_ and photosynthetic fluorescence parameters as the test endpoints showed that DSMO with a volume ratio of less than 2% would not affect the biomass and Photosynthesis of *Chlorella pyrenoidosa* within 96 h. All treatments were prepared in triplicate.

### 2.3. The Measurement of Photosynthetic Fluorescence Parameter

Photosystem II efficiency was estimated from the photosynthetic fluorescence parameters obtained using a fast repetition rate fluorometer (FRRf) (serial no. 16-0497-003; Chelsea Technologies Group Ltd., England). The fluorometer was equipped with Act2Run system software. An excitation light source was selected according to the absorption spectrum range of different algal species, and a 450-nm LED was selected for green algae. The whole measurement period lasted about 3 min. The samples were exposed to 8 sequential actinic light levels, increasing from 0 to 800 μmol·m^−2^·s^−1^. The relative electron transfer rate (rP) was calculated for each layer of photosynthetic light, and at same time a series of photosynthetic parameters were obtained [24,25].

*Fv/Fm* is the maximum photochemical quantum yield of PSII, which is considered to be related to water splitting and oxygen evolution [26]. The calculation formula is as follows:

(1) $$Fv/Fm=\left(Fm-F_{0}\right)/Fm$$

where *F*_0_ is the minimal fluorescence and *Fm* the maximum level of fluorescence measured after a dark adaptation.

*Yield* is the effective quantum yield of PSII photochemical energy conversion, which can be calculated as the following equation:

(2) $$Yield=\left(Fm^{\prime }-Ft\right)/Fm^{\prime }$$

where *Ft* and *Fm* are the steady-state level of fluorescence and the maximum level of fluorescence measured under ambient light, respectively.

*rP* is the relative PSII electron transfer rate, which is derived from the following equation:

(3) rP=Yield×E

where *E* is the incident photon irradiance. The fast light curve (FLCs) was constructed with irradiance (*E*) as abscissa and the relative PSII electron transfer rate (*rP*) as ordinate, and the initial slope α of the FLCs curve YIE was obtained. α represents the maximum light utilization coefficient, which is obtained by linear regression on the points in the light limited part of the FLC curve.

### 2.4. The Measurement of Biomass Parameter

Optical density (OD) is an effective indicator to replace the number of algae cells [27]. Previous studies found that OD_680_, as measured on a UV-2550 spectrophotometer (Shimadzu, Kyoto, Japan) at 680 nm, could be used to quantify the growth of *Chlorella* [28]. In the subsequent experiments, OD_680_ was used to represent the biomass of *C. pyrenoidosa*.

### 2.5. Data Processing and Statistical Analysis

We recorded the values of photosynthetic fluorescence parameters at each exposure time and analyzed the time trend of the parameters. One-way ANOVA was performed on the control group and the treatment group with the addition of different concentrations of toxicants to determine the impact of the toxicants. After one-way ANOVA, the least significant difference method (LSD) was used for post-multiple inspection, to obtain the lowest concentration that had a significant impact on the photosynthetic fluorescence parameters (*p* < 0.05). Statistical analyses were carried out using SPSS statistics 22.0.

The effect of toxicants on photosynthetic fluorescence parameters and OD_680_ was ultimately expressed as the inhibition rate or inhibition degree, calculated as follows:

(4) $$I_{t}\left(\%\right)=\frac{\left(u_{ct}-u_{kt}\right)}{u_{ct}}\times 100\%$$

where $u_{ct}$ and $u_{kt}$ indicate the values of the parameters of the control and treatment groups, where exposure time is *t*.

Dose response curves were constructed using a logistic mode, and the model formula is as follows:

(5) $$I\left(x\right)=\frac{A_{2}+\left(A_{1}-A_{2}\right)}{(1+\left(\frac{x}{X_{0}}){}^{P}\right)}$$

where *x* indicates concentration. *A*_1_, *A*_2_, *X*_0_, and *P* are constants. Through the dose-response curve, the median effective concentration, namely EC_50_, could be obtained.

## 3. Results

### 3.1. Rapid Toxicity Detection of Diverse Pollutants

**Figure 1:**
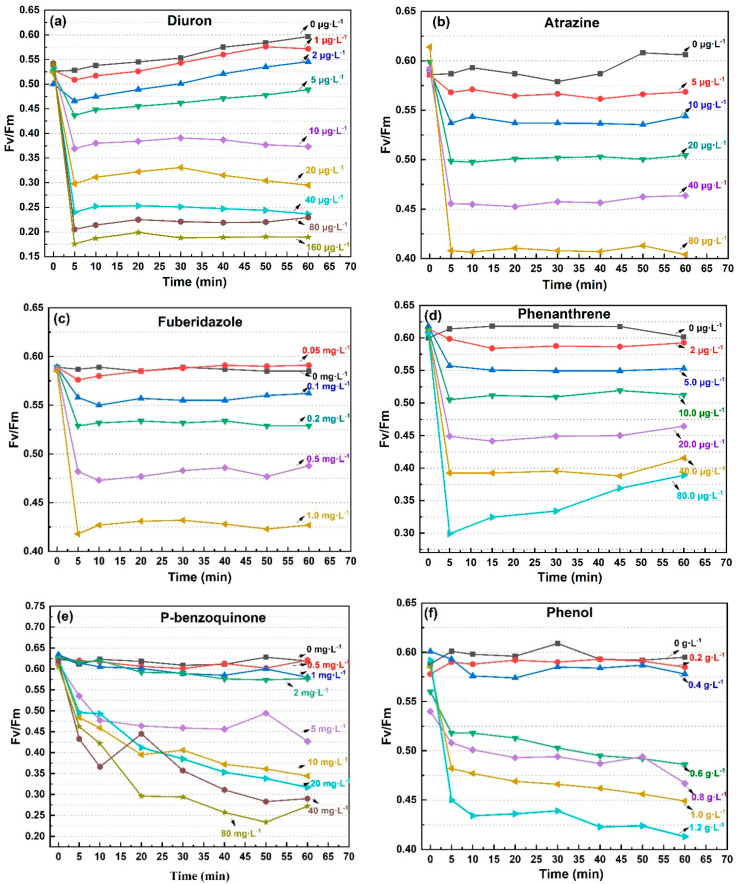

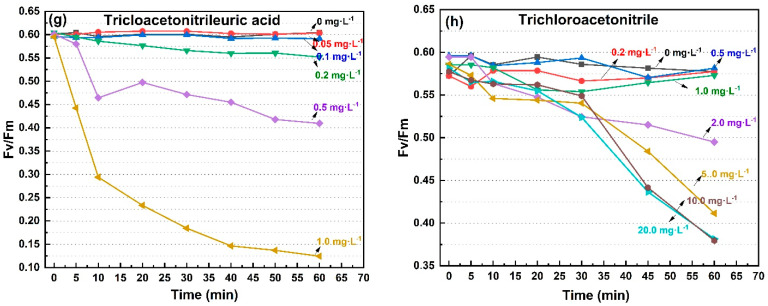
The Fv/Fm value trend of *C. pyrenoidosa* exposed to the 8 pollutants for 60 min. (**a**) diuron; (**b**) atrazine; (**c**) fuberidazole; (**d**) phenanthrene; (**e**) P-benzoquinone; (**f**) phenol; (**g**) trichloroacetonitrile uric acid; (**h**) trichloroacetonitrile. Fv/Fm—the maximum photochemical quantum yield of PSII.

Although the sensitivity of the four photosynthetic fluorescence parameters (Fv/Fm, Yield, α, rP) to pollutants was slightly different, their time response trends were consistent, represented by Fv/Fm, as shown in Figure 1. As herbicides, it was clear that diuron and atrazine had specific inhibitory effects on the electron transport in PSII [29]. However, the significant inhibitory effects of other types of pollutants on photosynthesis was also demonstrated in this study, such as fungicides, disinfectants, and polycyclic aromatic hydrocarbons. The photosynthetic fluorescence parameters began to decrease within a few minutes after exposure to the eight pollutants. In response to diuron, atrazine, fuberidazole, and phenanthrene, the photosynthetic fluorescence parameters of *C. pyrenoidosa* decreased rapidly within 5 min, but remained stable or even slightly recovered within 1 h, as shown in Figure 1a–d. In response to P-benzoquinone, phenol, trichloroacetonitrile uric acid, and trichloroacetonitrile, the photosynthetic fluorescence parameters of *C. pyrenoidosa* decreased within 1 h, but the rate of decline was not consistent. Judging by the degree to which the slope of the parameter’s decreasing curve tended to 0, most of the inhibition of the photosynthetic fluorescence parameters by phenol was observed within 5 min; and for P-benzoquinone and trichloroacetonitrile uric acid within 40 min, as shown in Figure 1e–g. For the response to trichloroacetonitrile, the obvious dose effect was manifested after 30 min, and the decrease in Fv/Fm within 60 min did not slow down, as shown in Figure 1h. From the above results, the decline rates of photosynthetic fluorescence parameters of *C. pyrenoidosa* exposed to various pollutants were different, but 1 h could be used as an appropriate toxicity response time. The eight pollutants mentioned above had rapid and obvious inhibitory effects on the photosynthetic fluorescence parameters (Fv/Fm, Yield, α, rP).

### 3.2. Response Sensitivity and Dose-Responses

**Figure 2:**
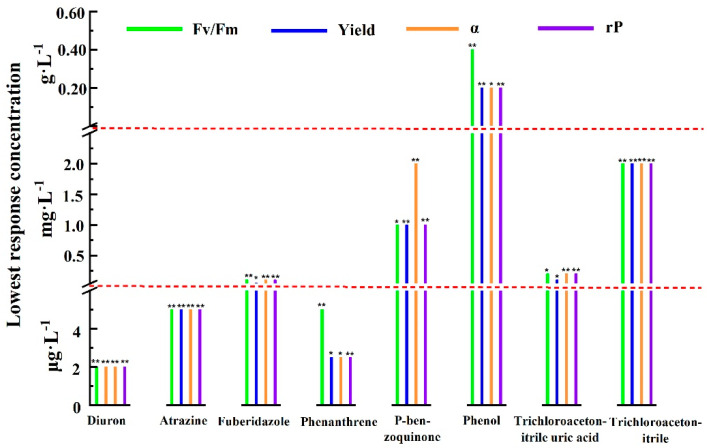
Within the experimental concentration range and when the exposure time was 1 h, the lowest concentration of different pollutants that had significant inhibitory effects on Fv/Fm, Yield, α, and rP (* 0.01 < *p* < 0.05; ** *p* < 0.01). Fv/Fm—the maximum photochemical quantum yield of PSII; Yield—the effective quantum yield of PSII photochemical energy conversion; α—maximum light utilization coefficient; rP—relative PSII electron transfer rate.

One-way ANOVA and post hoc multiple inspection were performed on all photosynthetic fluorescence parameter values after 1 h of exposure. Then we obtained the lowest concentration of pollutants that significantly inhibited the photosynthetic fluorescence parameters in the measured concentration range and took this concentration as the lowest response concentration of the parameters, as shown in Figure 2. The lowest response concentrations of the different photosynthetic fluorescence parameters for the same pollutant were the same or similar, and the numerical difference did not exceed an order of magnitude. As can be seen from Figure 2, the lowest response concentrations of photosynthetic fluorescence parameters to diuron, atrazine, fuberidazole, phenanthrene, P-benzoquinone, phenol, trichloroacetonitrile uric acid, and trichloroacetonitrile were 2 μg·L^−1^ (*p* < 0.05), 2 μg·L^−1^ (*p* < 0.05), 0.05 mg·L^−1^ (*p* < 0.05), 2 μg·L^−1^ (*p* < 0.05), 1.0 mg·L^−1^ (*p* < 0.05), 0.2 g·L^−1^ (*p* < 0.05), 0.1 mg·L^−1^ (*p* < 0.05), and 2.0 mg·L^−1^ (*p* < 0.05), respectively. The results showed that the photosynthetic fluorescence parameters were very sensitive to diuron and atrazine. Unexpectedly, phenanthrene, as a polycyclic aromatic hydrocarbon, was not reported to have a direct effect on the photosynthetic system, but the lowest response concentration of the photosynthetic fluorescence parameters to phenanthrene was very close to that of diuron. Although the above results were only the test results for one kind of algae, it would be facile to include more sensitive or ecologically important species by using the photosynthetic fluorescence parameters of algae for rapid detection of pollutants.

**Figure 3:**
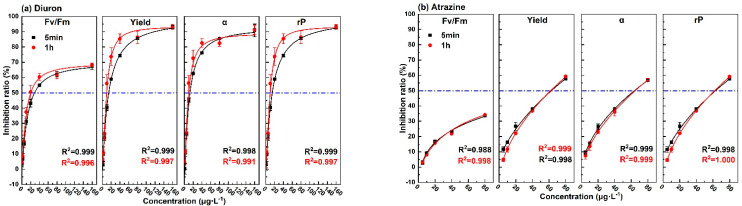

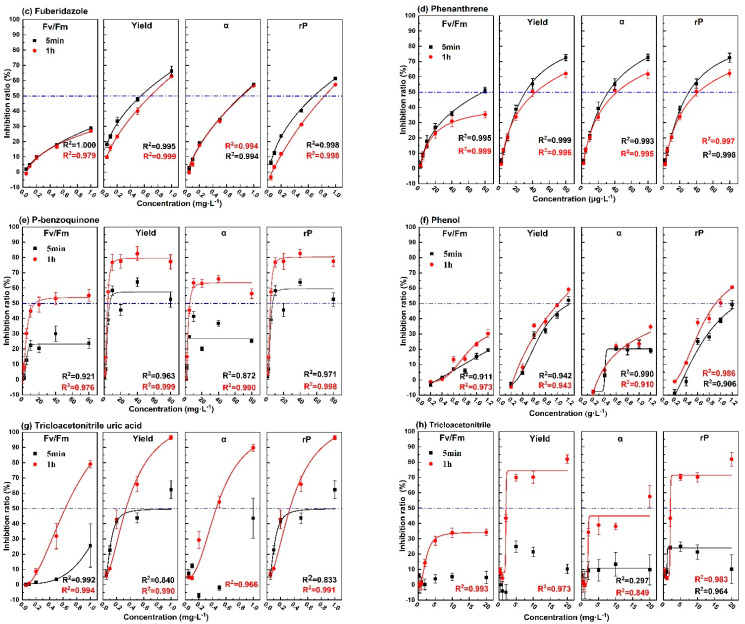
The dose–effect curve of the 8 pollutants on the photosynthetic fluorescence parameters (Fv/Fm, Yield, α, rP). (**a**) diuron; (**b**) atrazine; (**c**)fuberidazole; (**d**) phenanthrene; (**e**) p-benzoquinone; (**f**) phenol; (**g**) trichloroacetonitrile uric acid; (**h**) trichloroacetonitrile. Fv/Fm—the maximum photochemical quantum yield of PSII; Yield—the effective quantum yield of PSII photochemical energy conversion; α—maximum light utilization coefficient; rP—relative PSII electron transfer rate.

The changing trend of inhibition rate of photosynthetic fluorescence parameters (Fv/Fm, Yield, α, rP) with the concentration of the eight pollutants for the exposure times of 5 min and 1 h are shown in Figure 3. The 5 min dose–response curves of diuron, atrazine, fuberidazole, and phenanthrene were similar to those of 1 h. With the increase of concentration, the increase rate of the curves became slower, and the correlation coefficients were all above 0.99. For phenol, except for parameter α, the dose–response curves of the parameters at 5 min and 1 h were similar, and the correlation coefficient was more than 0.9. For p-benzoquinone, trichloroacetonitrile uric acid, and trichloroacetonitrile, within the same test concentration range, it can be clearly observed that the upper limit of the 1 h dose–effect curve was higher and the fitting effect was better. While for 5 min, not only the upper limit of the curve was low, sometimes it was even impossible to fit the curve. The above results show that for the five pollutants (diuron, atrazine, fuberidazole, phenanthrene, and phenol), the photosynthetic fluorescence parameters could be used to quantitatively evaluate the toxicity after 5 min of exposure. For the other three pollutants (p-benzoquinone, trichloroacetonitrile uric acid, trichloroacetonitrile), a quantitative evaluation could be achieved within 1 h. In the same concentration range, the upper limit of the inhibition rate of α, Yield, and rP were higher than Fv/Fm. This shows that a single photosynthetic fluorescence parameter Fv/Fm might cause an underestimation of aquatic toxicity.

### 3.3. Comparison with the Growth Inhibition Method

**Figure 4:**
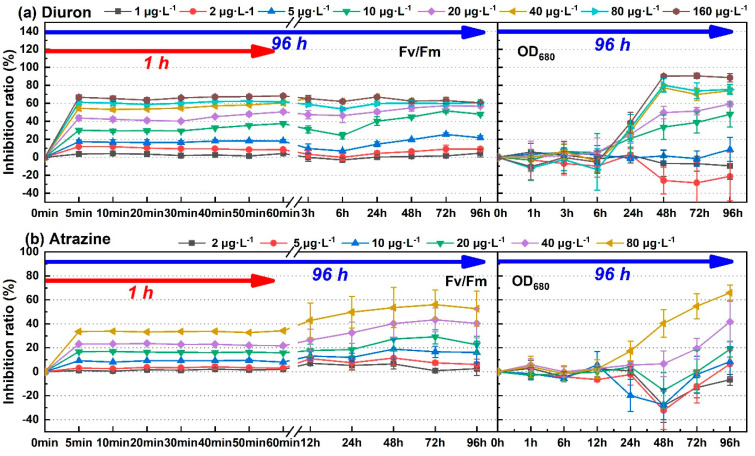

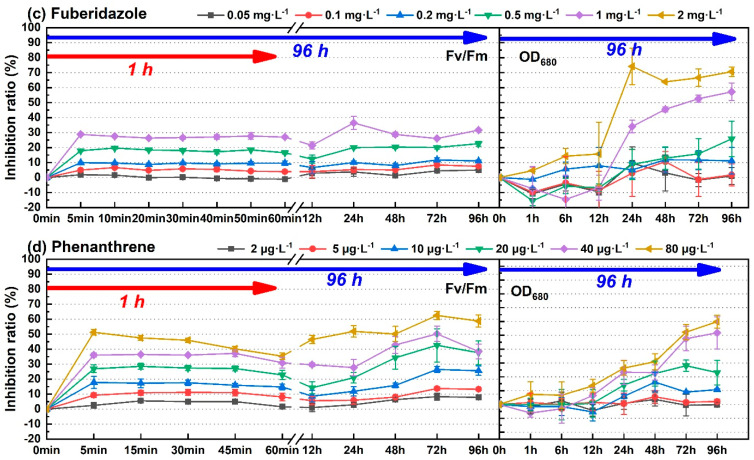
The time change of the inhibition rate of the four environmental pollutants on the photosynthetic fluorescence parameter Fv/Fm and the biomass parameter OD_680_ of *C. pyrenoidosa*. (**a**) Diuron; (**b**) atrazine; (**c**) fuberidazole; (**d**) phenanthrene. Fv/Fm—the maximum photochemical quantum yield of PSII; OD_680_—absorbance value of *C. pyrenoidosa* liquid at 680 nm.

The process of photosynthesis involves light absorption, electron transfer, photosynthetic phosphorylation, and carbon assimilation. Among them, carbon assimilation is the main source of biomass growth. In theory, the toxicity information obtained through the photosynthetic system was faster and more sensitive. Four pollutants, including diuron, atrazine, fuberidazole, and phenanthrene, were selected to measure the biomass parameter (OD_680_). One-way ANOVA and post hoc multiple inspection suggested that diuron, atrazine, fuberidazole, and phenanthrene, within the tested concentration range, began to significantly inhibit the growth of *C. pyrenoidosa* at 24 h, 24 h, 24 h, and 12 h, respectively (*p* ≤ 0.05). Figure 4 shows a comparison of the photosynthetic system and biomass inhibition results of *C. pyrenoidosa* exposed to the four environmental pollutants (diuron, atrazine, fuberidazole, and phenanthrene) within 96 h. According to the time effect analysis, the fluctuation of photosynthetic inhibition effect was not very strong, relative to the growth inhibition within the 96 h toxic exposure time, especially with a relatively stable state and a short time (1 h). The growth inhibition was a population-dependent phenomenon, which required time accumulation, and was obviously time-dependent. With the prolongation of exposure time, the growth inhibition rate had an upward trend. Taking diuron (10 μg·L^−1^) as an example, the relative standard deviation (RSD) of the short-term (5 min^−1^ h) and long-term (5 min–96 h) inhibition rates of Fv/Fm were 10.4% and 23.1%, respectively. The RSD of the inhibition rate of OD_680_ (24–96 h) was 32.1%. 

Since, photosynthetic inhibition eliminated the dependence on long-term exposure time, we directly compared the results of short-term (5 min, 1 h) photosynthetic inhibition and long-term (72 h, 96 h) growth inhibition, as shown in Table 2. It can be seen from Table 2, that for growth inhibition, the longer the exposure time, the lower the EC_50_ obtained. The EC_50_-96 h corresponding to diuron, atrazine, fuberidazole, and phenanthrene calculated according to the biomass parameter OD_680_ were 14.48 μg·L^−1^, 55.54 μg·L^−1^, 0.83 mg·L^−1^, and 43.81 μg·L^−1^, respectively. For photosynthetic inhibition, the difference between the parameters was obvious. The EC_50_ calculated by Fv/Fm was higher than the other three photosynthetic fluorescence parameters. Comparing the four photosynthetic fluorescence parameters (Fv/Fm, Yield, α, and rP), the lowest EC_50_-5 min corresponding to diuron, atrazine, fuberidazole, and phenanthrene were 12.74 μg·L^−1^, 62.94 μg·L^−1^, 0.55 mg·L^−1^, and 30.99 μg·L^−1^, respectively. While, the lowest EC_50_-1 h corresponding to diuron, atrazine, fuberidazole, and phenanthrene were 9.88 μg·L^−1^, 62.09 μg·L^−1^, 0.69 mg·L^−1^, and 42.37 μg·L^−1^, respectively. These results indicate that when EC_50_ was used to evaluate the toxicity of pollutants, and compared with traditional growth inhibition, photosynthetic fluorescence parameters as the endpoint of rapid toxicity detection did not cause an underestimation of toxicity. Compared with Fv/Fm, the toxicity data (EC_50_) obtained from the photosynthetic fluorescence parameters Yield, α, rP were lower and were more suitable as the end points of the biological toxicity test.

## 4. Discussion

The development of fast, simple, affordable, and ecologically-sound toxicity testing methods is very important for improving the supervision of harmful substances and realizing water safety early warning systems. As a test organism, microalgae could obtain many of the related test endpoints faster or easier, such as cell number, chlorophyll content, antioxidant system, oxygen production, and fluorescence emission, etc. Among them, cell number and chlorophyll content are closely related to indicators of algae growth and biomass, so the recording of toxic effects requires time to accumulate [23,30]. In this paper, OD_680_ was used to indicate the growth status of algae cells, and the results show that diuron, atrazine, atrazine, and phenanthrene began to significantly inhibit the growth of *C. pyrenoidosa* at 24 h, 24 h, 24 h, and 12 h, respectively. Moreover, the corresponding EC_50_ decreased with the prolongation of the toxicity exposure time. This conclusion was similar to the results of Shi Yajuan et al., in which *Chlorella* had obvious growth inhibition after 21 h of diuron stress, and the inhibition was significantly enhanced on the 2nd and 3rd days [31]. Changes at the cellular level usually do not require long-term accumulation and can also reflect the mechanism of toxic effects [32]. Jiang Lijuan et al. demonstrated the effects of Phe on the free radical content, antioxidant system, and lipid peroxidation products (MDA) of *Scenedesmus obliquus* and compared them with growth indicators [33]. The results proved that, although free radicals and antioxidant system indicators were considered to reflect the stress effects of organisms at the molecular level, they were not more sensitive than growth indicators. In addition, the detection of free radicals and related antioxidant indicators requires strict laboratory conditions and operations, which are time-consuming and labor-intensive. In the photosynthesis of phytoplankton, the release of oxygen, the fixation of C, and the change of chlorophyll fluorescence have always been considered to be related and could all be used as a measure of photosynthetic performance. Camuel et al. studied the effects of diuron and atrazine on the photosynthetic oxygen release rate of *Chlorella vulgaris* [15]. The results showed that the oxygen release rate could be used as the end point for rapid detection of the toxicity of diuron and atrazine. The EC_50_ corresponding to 20 min exposure were 72.26 μg·L^−1^ and 69.02 μg·L^−1^, respectively. This was similar to the results of this paper, using photosynthetic fluorescence parameters as the end point of the toxicity test, but the measurement parameters of the former study were generally single.

The fluorescence kinetics method applied to the measurement of phytoplankton aquatic toxicity has the characteristics of a simple measurement process, no sample pretreatment, and a non-invasive measurement process. Most of the previous related research focused on a single toxicant, and the time of exposure to the toxicants ranged from a few hours to a few days. Pérez et al. studied the effects of polycyclic aromatic hydrocarbons on the photosynthetic fluorescence parameters of *Isochrysis globosa* within 72 h [34]. Tiam et al. proved that photosynthetic fluorescence parameters could show the significant influence of pesticides on the photosynthetic system within 24 h of herbicide exposure [35]. The acute toxicity study on *Euglena agilis Carter* by Kottuparambil S et al. demonstrated that phenol significantly reduced the maximum quantum yield (Fv/Fm) and maximum photosynthetic electron transport rate (rPmax) of the photosystem (PSII) within 1 h [18]. However, from the results of this paper, the photosynthetic fluorescence parameters often changed within a few minutes of exposure to the toxicants, and 1 h was sufficient as an appropriate toxic exposure time; a longer exposure time did not have an advantage. Among the series of photosynthetic fluorescence parameters, the most commonly used in toxicity tests is Fv/Fm [36,37]. However, this paper showed Fv/Fm was not the optimal toxicity test endpoint. It can be seen from Table 2 that among the four photosynthetic fluorescence parameters, only the EC_50_ obtained by Fv/Fm was higher than that obtained by OD_680_. Moreover, the dose–effect results of phenanthrene and diuron show that the EC_50_ obtained from Fv/Fm was approximately twice that of α, rP, and Yield. Therefore, more consideration should be given to the application of the parameters α, rP, and Yield in toxicity testing.

## 5. Conclusions

Photosynthesis is the most important physiological process in phytoplankton and is the basis of its growth. Any inhibition of its growth should be preferentially reflected in the photosynthetic process. Rapid detection of eight common pollutants showed that the inhibitory effect of the pollutants could be reflected in the photosynthetic fluorescence parameters (Fv/Fm, yield, α, rP) within a few minutes. Moreover, the method of photosynthetic inhibition was not unique to direct PSII inhibitors (diuron and atrazine), and it could also achieve rapid toxicity detection for pollutants not directly acting on the photosystem II. Diuron, atrazine, fuberidazole, and phenanthrene were selected for a comparison of photosynthetic inhibition and growth inhibition. Compared with the 3–4 day growth inhibition method, the photosynthetic inhibition method had a considerable time saving advantage, which provided a new method for the emergency monitoring of water pollution accidents and the rapid assessment of ecological environment risk. A bioassay method based on the rapid changes of photosynthetic fluorescence parameters has the most potential for field testing. This study did not involve the study of algae species differences. However, in the future, a variety of test objects could be customized according to the ecosystem or interested groups, to study the impact of toxic pollutants on a set of algae species, so as to provide a more ecologically representative response.

## Figures and Tables

**Table 1:** Toxicants tested and solvents used, practical application, test index, exposure time, exposure concentration range, and source of purchase.

Pollutants/Solvent	Applications	Test Index	Exposure Time		Con. Range Tested	Source of Purchase
			Shortest	Longest		
Diuron/DSMO	Herbicide	Fv/Fm, Yield, α, rP, OD_680_	5 min	96 h	1–160 μg·L^−1^	Aladdin (Shanghai, China), 99% pure
Atrazine/DSMO		Fv/Fm, Yield, α, rP, OD_680_	5 min	96 h	2–80 μg·L^−1^	TCI Shanghai, >97% pure
Fuberidazole/DSMO	Fungicide	Fv/Fm, Yield, α, rP, OD_680_	5 min	96 h	0.05–1 mg·L^−1^	Aladdin (Shanghai, China)
Phenanthrene/DSMO	Organic chemical raw materials	Fv/Fm, Yield, α, rP, OD_680_	5 min	96 h	2–80 μg·L^−1^	Aladdin (Shanghai, China), 97% pure
P-benzoquinone/DSMO		Fv/Fm, OD Yield, α, rP	5 min	1 h	0.5–80 mg·L^−1^	Aladdin (Shanghai, China), 97% pure
Phenol/water		Fv/Fm, Yield, α, rP	5 min	1 h	0.2–1.2 g·L^−1^	Aladdin (Shanghai, China), AR
Tricloacetonitrile uric acid /DSMO	Disinfectant bleach	Fv/Fm, Yield, α, rP	5 min	1 h	0.05–1 mg·L^−1^	Aladdin (Shanghai, China), 98% pure
Trichloroacetonitrile/DSMO	Disinfection by-products	Fv/Fm, Yield, α, rP	5 min	1 h	0.2–20 mg·L^−1^	Aladdin (Shanghai, China), 97% pure

Fv/Fm—the maximum photochemical quantum yield of PSII; Yield—the effective quantum yield of PSII photochemical energy conversion; α—maximum light utilization coefficient; rP—relative PSII electron transfer rate; OD_680_—absorbance value of *C. pyrenoidosa* liquid at 680 nm.

**Table 2:** *C. pyrenoidosa* exposed to 4 toxic pollutants: EC_50_ and its confidence interval (95%) for photosynthetic inhibition (Fv/Fm, Yield, α, and rP) and growth inhibition (OD_680_).

Toxicants	Photosynthetic Inhibition					Growth Inhibition	
		Fv/Fm	Yield	α	rP		OD_680_
Diuron	EC_50_-5 min	28.72 μg·L^−1^ (26.47–31.08)	14.51 μg·L^−1^ (13.73–15.17)	12.74 μg·L^−1^ (11.98–13.57)	14.53 μg·L^−1^ (13.73–15.32)	EC_50_-72 h	19.56 μg·L^−1^ (13.41–27.74)
	EC_50_-1 h	20.16 μg·L^−1^ (15.01–28.06)	9.94 μg·L^−1^ (8.00–12.78)	9.88 μg·L^−1^ (7.05–15.96)	9.94 μg·L^−1^ (8.0–12.94)	EC_50_-96 h	14.48 μg·L^−1^ (8.62–18.19)
Atrazine	EC_50_-5 min	-	62.94 μg·L^−1^	63.85 μg·L^−1^ (49.97–77)	63.10 μg·L^−1^	EC_50_-72 h	74.81 μg·L^−1^
	EC_50_-1 h	-	62.09 μg·L^−1^ (51.62–76.40)	64.75 μg·L^−1^ (44.41–74.82)	62.02 μg·L^−1^ (55.68–67.69)	EC_50_-96 h	55.54 μg·L^−1^ (31.90–74.22)
Fuberidazole	EC_50_-5 min	-	0.55 mg·L^−1^	0.82 mg·L^−1^	0.69 mg·L^−1^ (0.34–0.92)	EC_50_-72 h	0.94 mg·L^−1^
	EC_50_-1 h	-	0.69 mg·L^−1^ (0.40–0.91)	0.82 mg·L^−1^ (0.091–0.97)	0.84 mg·L^−1^ (0.57–0.94)	EC_50_-96 h	0.83 mg·L^−1^ (0.57–1.12)
Phenanthrene	EC_50_-5 min	77.54 μg·L^−1^	31.32 μg·L^−1^ (26.08–38.42)	32.08 μg·L^−1^ (27.25–37.95)	30.99 μg·L^−1^ (26.39–37.41)	EC_50_-72 h	53.39 μg·L^−1^
	EC_50_-1 h	-	43.93 μg·L^−1^ (30.43–60.30)	42.62 μg·L^−1^ (29.34–66.04)	42.37 μg·L^−1^ (32.21–55.49)	EC_50_-96 h	43.81 μg·L^−1^ (25.62–72.16)

EC_50_—the median effective concentration; OD_680_—absorbance value of *C. pyrenoidosa* liquid at 680 nm; Fv/Fm—the maximum photochemical quantum yield of PSII; Yield—the effective quantum yield of PSII photochemical energy conversion; α—maximum light utilization coefficient; rP—relative PSII electron transfer rate.

## Data Availability

Not applicable.
